# Comparative transcriptome analysis of *Glyphodes pyloalis* Walker (Lepidoptera: Pyralidae) reveals novel insights into heat stress tolerance in insects

**DOI:** 10.1186/s12864-017-4355-5

**Published:** 2017-12-19

**Authors:** Yuncai Liu, Hang Su, Rongqiao Li, Xiaotong Li, Yusong Xu, Xiangping Dai, Yanyan Zhou, Huabing Wang

**Affiliations:** College of Animal Sciences, Zhejiang University, Hangzhou, 310058 China

**Keywords:** *Glyphodes pyloalis*, Heat stress, RNA-Seq, Heat shock protein, Redox homeostasis, Metabolism, Immune

## Abstract

**Background:**

Heat tolerance is a key parameter that affects insect distribution and abundance. *Glyphodes pyloalis* Walker (Lepidoptera: Pyralidae) is a devastating pest of mulberry in the main mulberry-growing regions and can cause tremendous losses to sericulture by directly feeding on mulberry leaves and transmitting viruses to *Bombyx mori*. Moreover, *G. pyloalis* shows a prominent capacity for adaptation to daily and seasonal temperature fluctuations and can survive several hours under high temperature. To date, the molecular mechanism underlying the outstanding adaptability of this pest to high temperature remains unclear.

**Results:**

In this study, we performed comparative transcriptome analyses on *G. pyloalis* exposed to 25 and 40 °C for 4 h. We obtained 34,034 unigenes and identified 1275 and 1222 genes significantly upregulated or downregulated, respectively, by heat stress. Data from the transcriptome analyses indicated that some processes involved in heat tolerance are conserved, such as high expression of heat shock protein (HSP) genes and partial repression of metabolism progress. In addition, vitamin digestion and absorption pathways and detoxification pathways identified here provided new insights for the investigation of the molecular mechanisms of heat stress tolerance. Furthermore, transcriptome analysis indicated that immune and phosphatidylinositol signaling system have a close relationship with heat tolerance. In addition, the expression patterns of ten randomly selected genes, such as HSP and cytochrome P450, were consistent with the transcriptome results obtained through quantitative real-time PCR.

**Conclusions:**

Comparisons among transcriptome results revealed the upregulation of HSPs and genes involved in redox homeostasis, detoxication, and immune progress. However, many metabolism progresses, such as glycolysis/gluconeogenesis and fatty acid biosynthesis, were partially repressed**.** The results reflected that the heat tolerance of *G. pyloalis* is a fairly complicated process and related to a broad range of physiological regulations. Our study can improve understanding on the mechanisms of insect thermal tolerance.

**Electronic supplementary material:**

The online version of this article (10.1186/s12864-017-4355-5) contains supplementary material, which is available to authorized users.

## Background

High temperature is an environmental factor that limits the distribution and abundance of insects. Insects are sensitive to temperature changes [1]. Over the past 30 years, global warming has led to significant changes in the number of insect species [2]. Several insect species, such as *Drosophila melanogaster* (*D. melanogaster*) and *Bombyx mori* (*B. mori*), are sensitive to heat stress, and the mass mortalities of these insects are often caused by high temperature [3, 4]. Conversely, many pests have evolved outstanding capability to adapt to high-temperature stimulations [5]. In recent years, many studies have focused on the effect of temperature on these insects because of their adaptability to a broad range of temperature [6].

Heat stress and acclimation of insect are currently considered a multistep process, involving a combination of behavioral, physiological, and cellular responses [7, 8]. Recent studies showed that heat tolerance in certain organisms is related to protein folding, degradation, transport, and metabolism [9, 10]. Moreover, current studies on flies revealed that several signal pathways, such as stress-responsive c-Jun N-terminal kinase signaling pathway, play an important role in adaptive metabolic response to heat stress [11]. Thus, many biologists have attempted to uncover the mechanisms underlying gene expression regulation under heat stress. Recently, researchers have characterized proteomic responses induced by heat stress in insects, such as *Aphids*, *D. melanogaster*, and *B. mori*. These studies displayed many protein-determining thermotolerances, such as chromatin remodeling and translation, iron ion and cell redox homeostasis, and carbohydrate and energy metabolism [12–14]. Despite these findings, minimal information is available on the gene expression profile of pest resistant to heat stress.

*Glyphodes pyloalis* Walker (Lepidoptera: Pyralidae), a specialist pest on mulberry, is widely distributed throughout Asia. This pest can damage sericulture not only by feeding on mulberry but also by transmitting viruses to *B. mori*. *G. pyloalis* encounters a wide range of daily and seasonal temperature fluctuations. On a summer day, the average temperature in Hangzhou (120.2′ E, 30.3′ N), Zhejiang Province, China is about 33.8 °C, and the highest temperature can reach almost 42 °C. Temperature increases during the summer lead to adverse effects on temperate bivoltine silkworm rearing and cause economic losses. In contract, *G. pyloalis* adapt to relatively high temperatures (35–40 °C) [15]. However, previous studies of *G. pyloalis* have focused only on single genes, such as *α-* and *β-glucosidase* [16]; the molecular mechanisms involved in the heat responses have not been explored through the transcriptome method, which has been widely used in profile gene expressions in insects. Uncovering the conundrum of the heat tolerance of *G. pyloalis* through RNA-Seq technique is reasonable, because the mechanism of *G. pyloalis* in managing heat stress remains unknown.

Midgut is the primary site of digestion and absorption in insects [17]. This site not only controls food storage and nutrient absorption but also maintains water, ion, and osmotic pressure balance [18]. Moreover, the midgut of an insect is involved in immunity and detoxication of harmful substances during digestion and absorption [19]. Thus, it is a pivotal organ for the interaction between an insect and external environment, and it represents a key target for the heat tolerance of an insect. However, few studies have focused on midgut transcriptome response to high temperature [20].

In the present study, *G. pyloalis*, an important lepidopteran pest, was assessed for the effects of heat acclimation. The results showed that approximately one-seventh of the transcriptome are differentially regulated upon heat acclimation. In addition, our results revealed that genes, which are related to heat tolerance, received pronounced effect after heat stimulation. This result showed that heat tolerance is a more complex process than we previously assumed. Our study can improve understanding on the molecular mechanism of heat tolerance in insects and provide novel targets for pest prevention and control.

## Results

### Heat resistance of *G. pyloalis* Walker

**Fig. 1:**
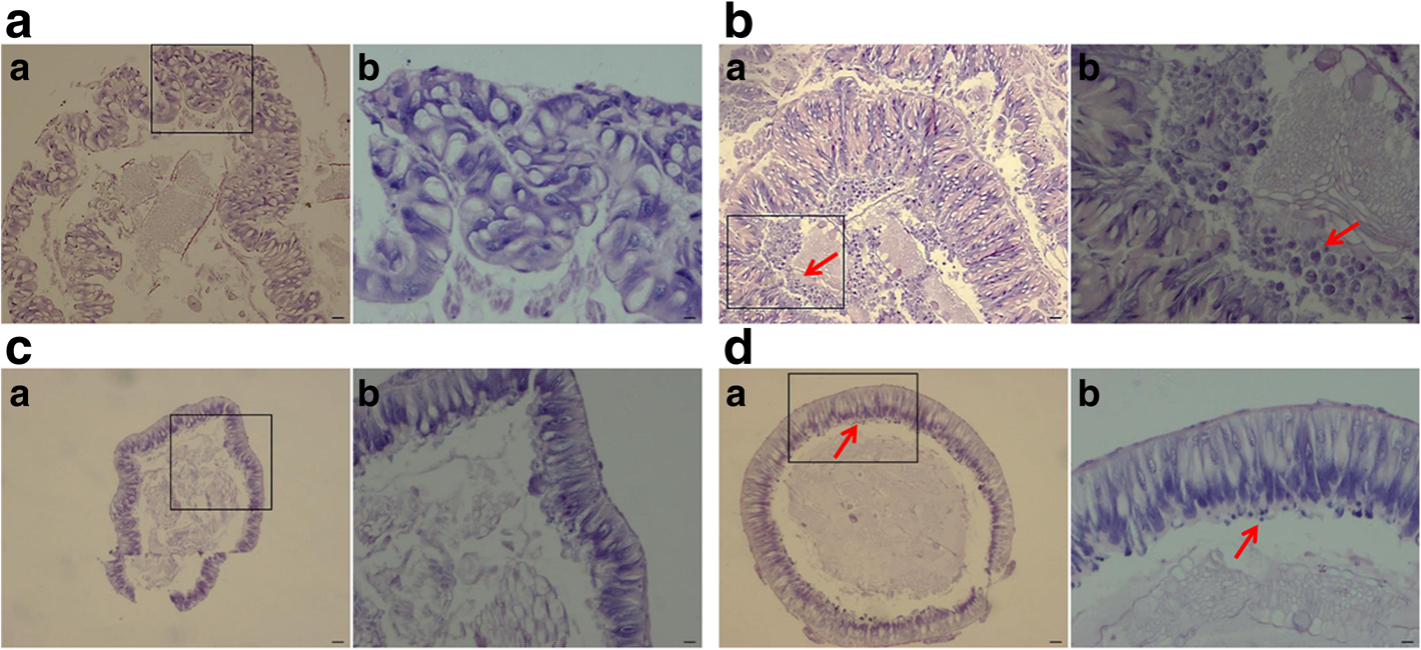
Morphological changes of midgut with different treatments. Morphological changes of *B. mori* (A–B) and *G. pyloalis* (C–D) were observed. Each (a) is a picture with low magnification, and the blank-lined area is shown in (b) with high magnification. Minimal bubble-like structure was observed in *G. pyloalis* after 4 h of heat treatment. However, many bubble-like structures (indicated by arrowheads) were produced in *B. mori*. Bars: A–a, B–a, C–a, and D–a: 10 μm; A–b, B–b, C–b, and D–a: 3.3 μm

*G. pyloalis* and *B. mori* are specialist insects on mulberry. Morphological changes in the midguts of *G. pyloalis* and *B. mori* were monitored after heat stress. Histological staining was performed to determine whether *G. pyloalis* can elicit heat resistance in its histomorphology and structure level under high temperatures (Fig. 1). After exposure to 40 °C for 4 h, the midgut of *B. mori* had a weaker condition than that exposed at 25 °C (Fig. 1a and b). Heat stress resulted in significant changes, and a large number of bubble-like structures were observed adjacent to the midgut contents of *B. mori* (Fig. 1b). However, structures observed in the midgut of *G. pyloalis* were fewer than those in *B. mori* after heat stress (Fig. 1b and d).

### mRNA sequencing, assembly, and functional annotation

**Fig. 2:**
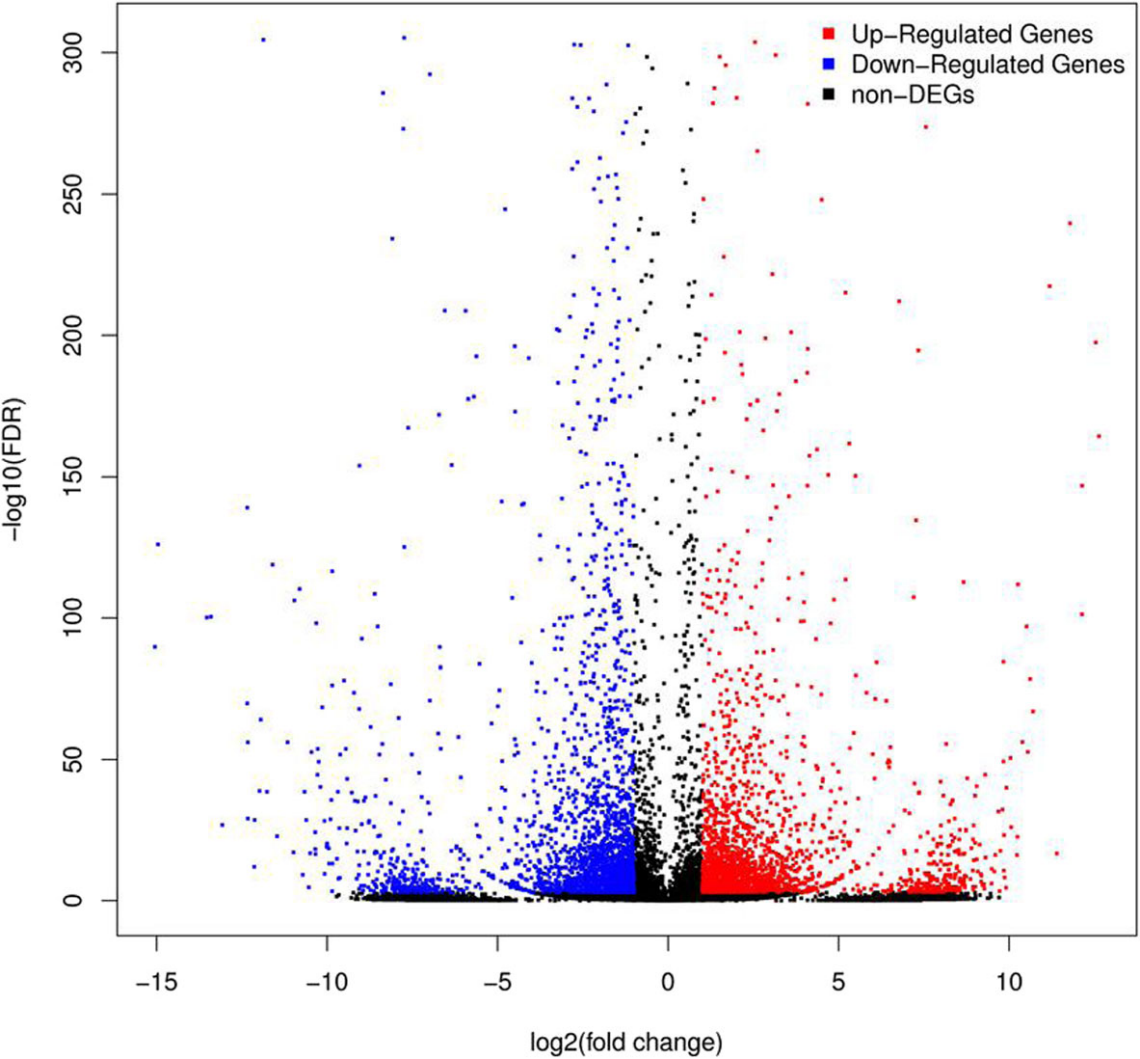
DEGs in the midgut after different temperature treatments. The variability pattern was displayed by volcano plot. Y axis represents −log10 significance. X axis represents log2-fold change. Red points represent upregulated genes. Blue points represent downregulated genes based on the discriminative significance values (|fold change| ≥ 4.0 and FDR ≤ 0.001) adopted in this study

We performed RNA-Seq to quantify the expression of genes in *G. pyloalis* to elucidate the molecular basis of heat stress response. RNA was extracted from the midgut of *G. pyloalis* and the transcriptome was sequenced using Illumina short reads. Approximately 13.37 Gb data and 34,034 unigenes were obtained (Additional file 1). The total length, average length, N50, and GC content of unigenes were 36,437,658 bp; 1070 bp; 2000 bp; and 43.09%, respectively (Table 1). We also detected the distinction of unigene length distributions between the two treatments and found no significant difference (Additional file 2). However, there were 22,729 unigenes expressed in the normal condition (25 °C) while 23,051 unigenes expressed after heat stress. We then annotated our unigenes with seven functional databases and found that 19,653 (57.75%), 13,170 (38.70%), 15,353 (45.11%), 8354 (24.55%), 15,546 (45.68%), 3878 (11.39%), and 14,410 (42.34%) unigenes can be mapped to NR, NT, UniProtKB/Swiss-Prot (Swiss-Prot), Cluster of Orthologous Groups of proteins (COG), Kyoto Encyclopedia of Genes and Genomes (KEGG), Gene Ontology (GO), and Interpro databases, respectively. After performing functional annotation, we detected 19,609 CDS. The remaining unaligned unigenes were analyzed using ESTScan to predict the coding regions. Another 1821 CDS were obtained after the analysis. The *G. pyloalis* sequences showed 42.9% matches with *B. mori*, followed by *Danaus plexippus* (27.63%), *Papilio xuthus* (2.2%), and *Riptortus pedestris* (2.13%) (Additional file 3). Furthermore, 2497 unigenes were differentially regulated after heat shock treatment with a criterion of |fold change| ≥4.0 and FDR ≤ 0.001 in DEGs definition. Of these DEGs, 1275 genes were upregulated, and 1222 genes were downregulated (Fig. 2). Ten significantly most upregulated genes that response to heat stress are as follows: ribonuclease, thiolase 4, peroxisomal multifunctional enzyme type 2-like, keratin-associated protein 10–7-like, aldehyde dehydrogenase isoform 1, PGI4-45_10 phosphoglucose isomerase, Probable cytochrome P450 304a1, Purine nucleoside phosphorylase associated with the functions redox homeostasis, detoxication and many metabolisms progresses (Additional file 4). This analysis showed heat stress had significant effect on the gene expression.

**Table 1:** Distribution of differentially expressed unigenes and Quality metrics of Unigenes

Sample	Total Number	Total Length	Mean Length	N50	N70	N90	GC (%)
Control	27,703	27,143,070	979	1789	987	365	43.42
Heat-shock	29,106	27,433,438	942	1693	929	353	43.34
All-Unigene	34,034	36,437,658	1070	2000	1146	397	43.09

N50: 50% of the Total Length is contained in Unigenes greater than or equal to this value

### GO and KEGG analyses of DEGs

**Fig. 3:**
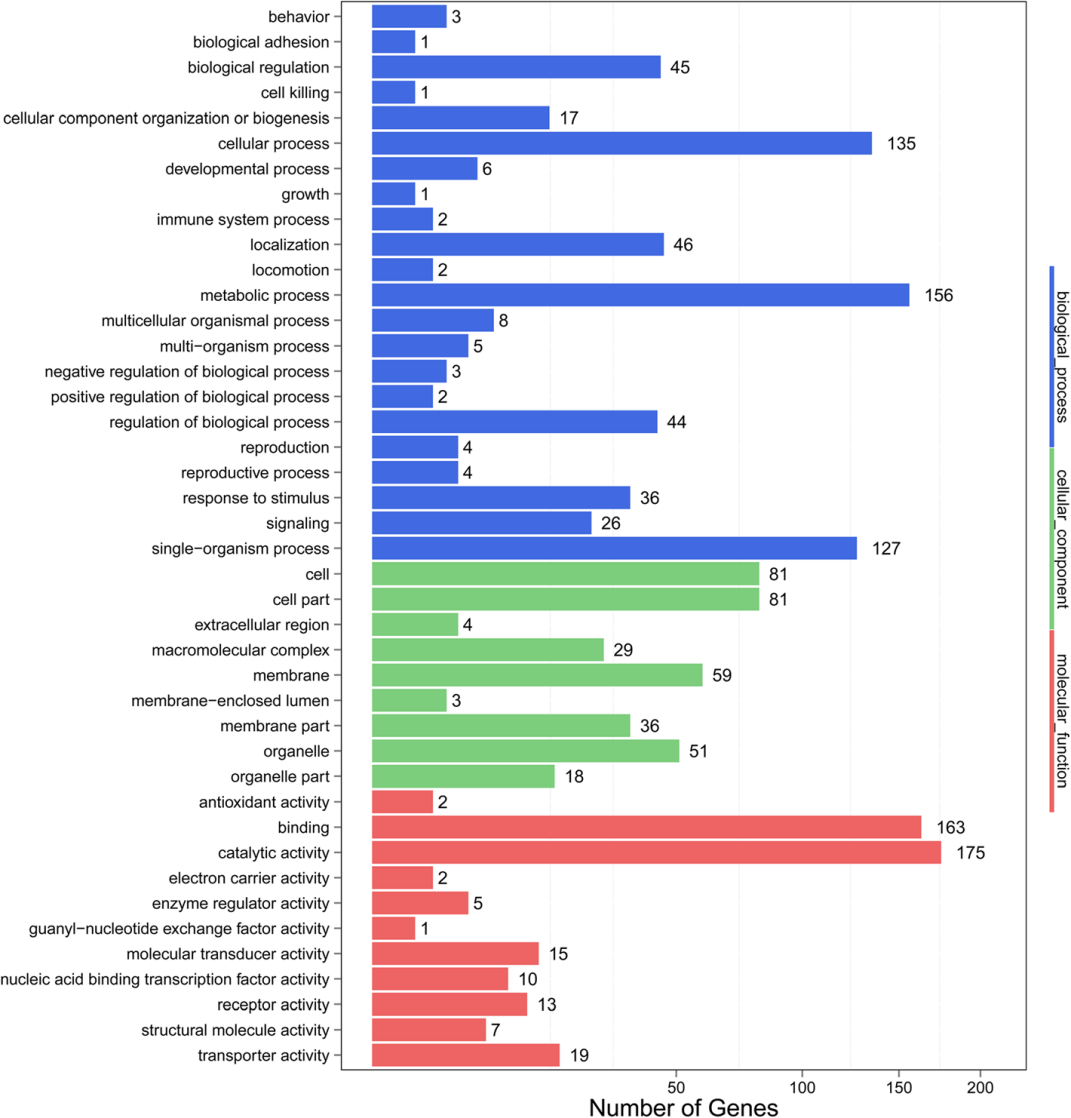
GO enrichment analysis revealed the biological processes most associated with detected DEGs. Based on the GO results, cellular progress, metabolic progress, binding, and catalytic activity were the most enriched GO terms under heat stress

We focused on the 1275 induced and 1222 repressed genes to further understand the overall biology of transcriptional response in *G. pyloalis* to heat stress. For GO analysis, we annotated DEGs into three GO categories, namely, cell component, molecular function, and biological process (Fig. 3). In the cell component category, the terms “glutamyl-tRNA amidotransferase complex” and “membrane” were the enriched components. “Steroid hormone receptor activity” and “lipid transporter activity” were the top two molecular function terms. The most enriched components of the biological process category were “steroid hormone mediated signaling pathway”, “response to a steroid hormone” and “cellular response to steroid hormone stimulus”.

KEGG is a database for biological systems that integrates genomic, chemical, and systemic functional information [21]. In this study, 1666 DEGs were mapped to 291 pathways. Among these DEGs, “metabolic pathways” was the most dominant pathway in the midgut. In addition, many pathways, such as “sphingolipid metabolism”, “phenylalanine metabolism”, “RIG-I-like receptor signaling pathway” and “Toll-like receptor signaling pathway” were also enriched (Additional file 5). These results indicated an integration between metabolic and immune responses during heat tolerance, and this integration ensures energy balance and permits growth and defense in *G. pyloalis*.

### Expression of pathways and genes for heat tolerance

#### Heat shock proteins (HSPs)

**Fig. 4:**
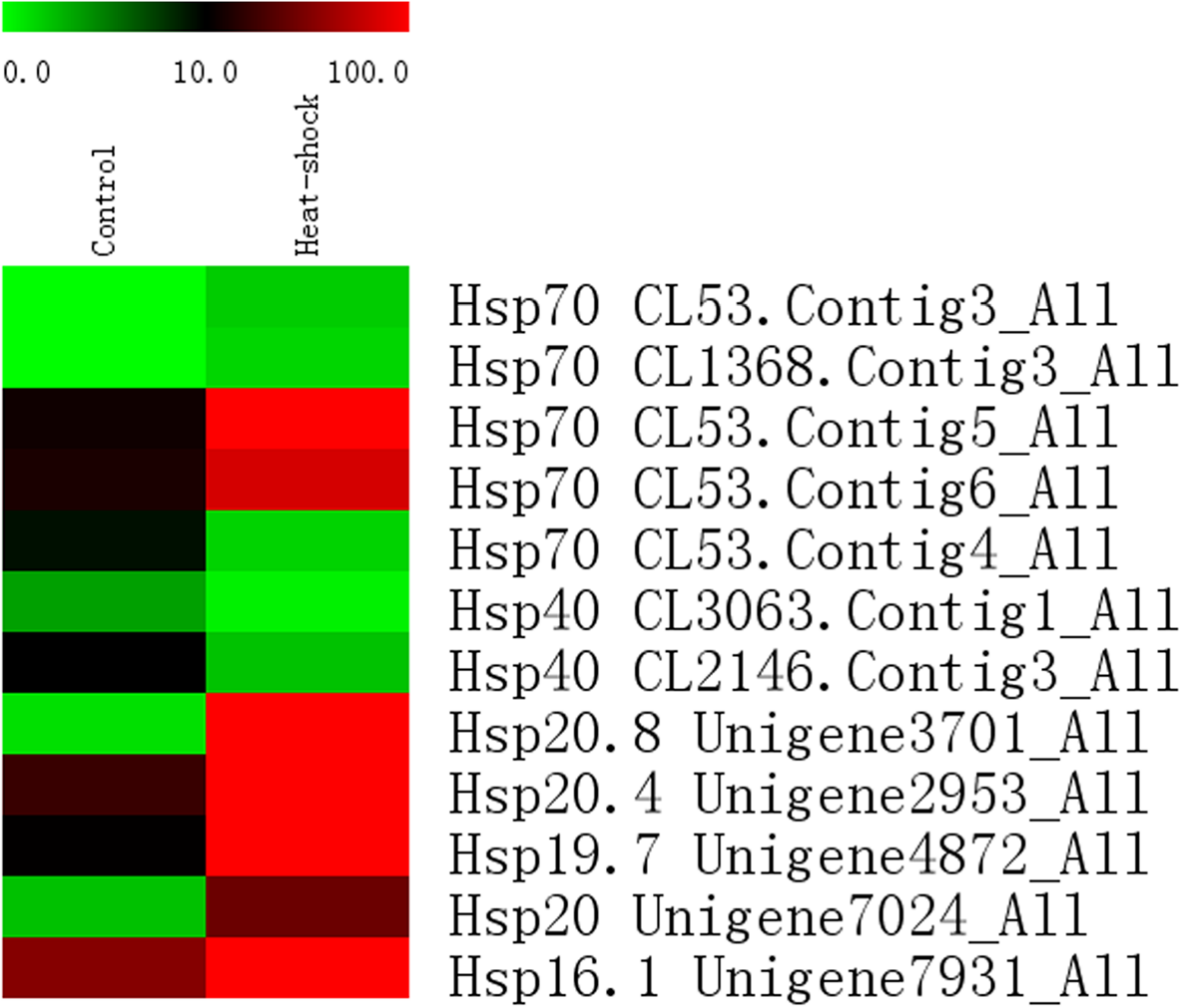
Comparative distribution of HSP coding genes between two samples. High temperature resulted in gene expression related to HSP70, HSP40, and small HSP. The color scale is displayed at the upper left, which encompasses from the lowest (green) to the highest (red) RPKM value

The transcriptional response of insects to heat shock includes a large number of HSPs, implying a universal mechanism of heat tolerance in insects [22]. We focused on genes that encoded HSPs to reveal the generality and particularity of heat tolerance between *G. pyloalis* and other insects. In this study, the data revealed upregulation or downregulation of HSPs under heat stress conditions. As shown in Fig. 4, 12 HSPs, including HSP70, HSP40, and small HSPs (sHSPs), which were distributed in different families, were identified (|fold change| ≥ 4.0 and FDR ≤ 0.001). Among these HSPs, five genes belonged to the HSP70 family, whereas two and five genes belonged to the HSP40 and sHSP, respectively. In terms of gene expression levels, nine HSPs displayed greater than fourfold increased expression, indicating a vital role in protein processing and heat tolerance in *G. pyloalis*.

### Antioxidant and detoxication

**Fig. 5:**
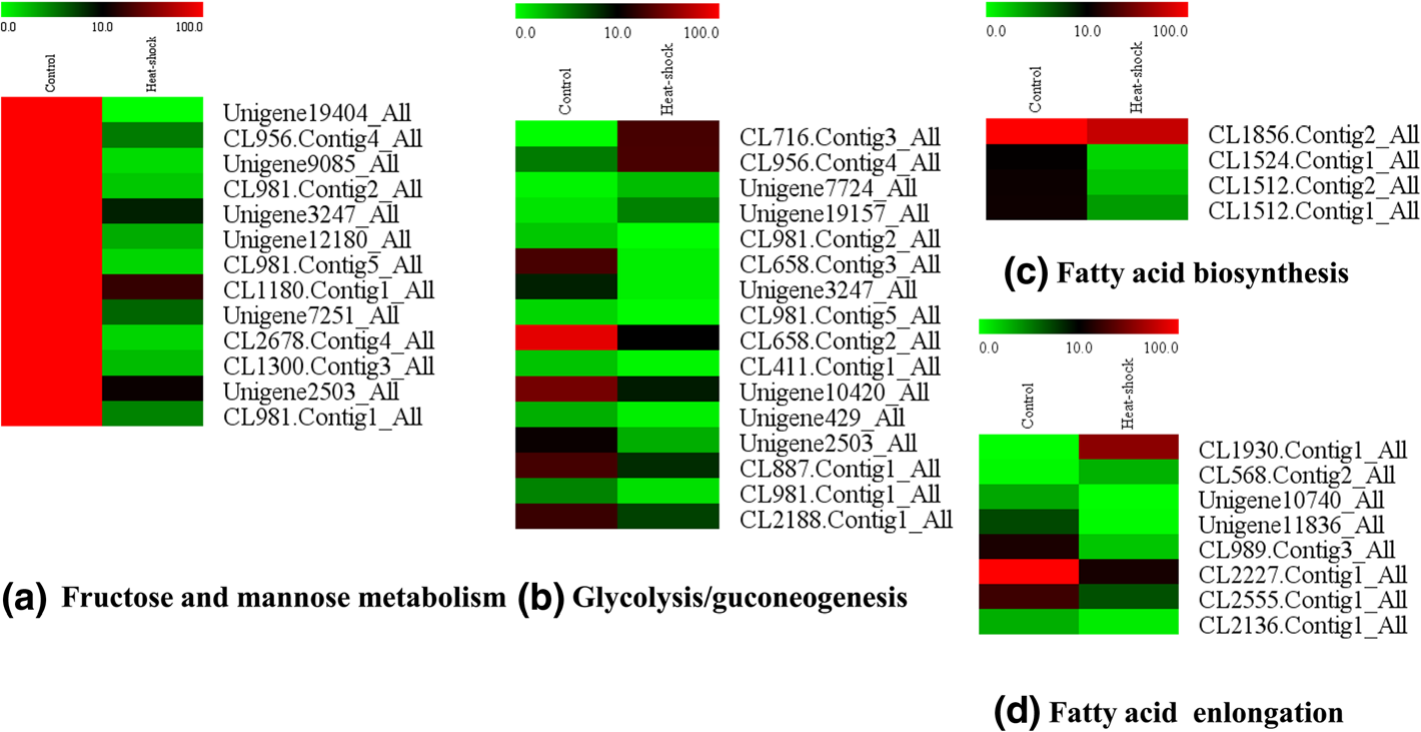
Heatmap of the expression level of different pathways. Expression level of some genes of fructose and mannose metabolism, glycolysis/gluconeogenesis, fatty acid biosynthesis, and fatty acid elongation was shown in **a**, **b**, **c**, and **d**, respectively. The color scale is shown at the upper left, which encompasses from the lowest (green) to the highest (red) RPKM value

Apart from genes encoding HSPs, several genes, which are involved in antioxidant and detoxication and regulated by heat stress, were identified. These genes included genes encoding two superoxide dismutase (Unigene21826_All and CL573.Contig1_All), a peroxidase (CL1988.Contig2_All), and a thioredoxin (CL1904.Contig1_All). In addition, these genes are directly linked to the generation of reactive oxygen species (ROS) under heat stress [23, 24] (Table 2). Detoxication-related genes were also identified in our study, including genes encoding glutathione S-transferase (CL2634.Contig1_All, Unigene19946_All, Unigene21727_All, and CL3231.Contig2_All) and cytochrome P450s (CYPs). 14 of the 23 cytochrome P450 (CYPs) unique sequences were induced, and most of these sequences were grouped into CYP3 clade (five of seven sequences), CYP6 clade (four of eight unique sequences), and CYP9 (three sequences; Fig. 5). Meanwhile, the polypeptides of the two unique sequences, namely, CL716.Contig3_All and Unigene19157_All, were determined as unigenes for aldehyde dehydrogenase (ALDH). They have been considered effective detoxifying enzymes and are involved in many fundamental biochemical pathways, including reduced cytotoxic aldehydes triggered by lipid peroxidation [25]. Therefore, antioxidant and detoxication have a close relationship with heat tolerance and even share a similar handling mechanism.

**Table 2:** Changes in the transcriptional expression of redox homeostasis and detoxication-related genes in response to thermal stress (40 °C) for 4 h

Gene ID	Control	Heat shock	Regulated	*P* value	Gene description
CL1904.Contig1_All	1.66	83.63	up	0	Thioredoxin
Unigene21826_All	0.29	4.95	up	7.98E-09	[Cu-Zn] SOD
CL573.Contig1_All	13.58	57.84	up	3.75E-84	[Cu-Zn] SOD
CL1988.Contig2_All	2.37	16.18	up	6.73E-122	POD
CL2634.Contig1_All	0.01	3.84	up	1.45E-08	GSTT1
Unigene19946_All	0.01	2.78	up	4.64E-06	GSTe4
Unigene21727_All	0.17	3.37	up	1.24E-05	GSTu1
CL3231.Contig2_All	1.05	4.24	up	1.16E-13	GSTT1
CL716.Contig3_All	0.01	35.38	up	2.09E-242	ALDH
Unigene19157_All	1.04	4.91	up	2.02E-05	ALDH

Significant FDR ≤ 0.001 for mRNA expression is represented. SOD: Superoxide dismutase; ALDH: Aldehyde dehydrogenase

### Metabolism and protein turnover

**Fig. 6:**
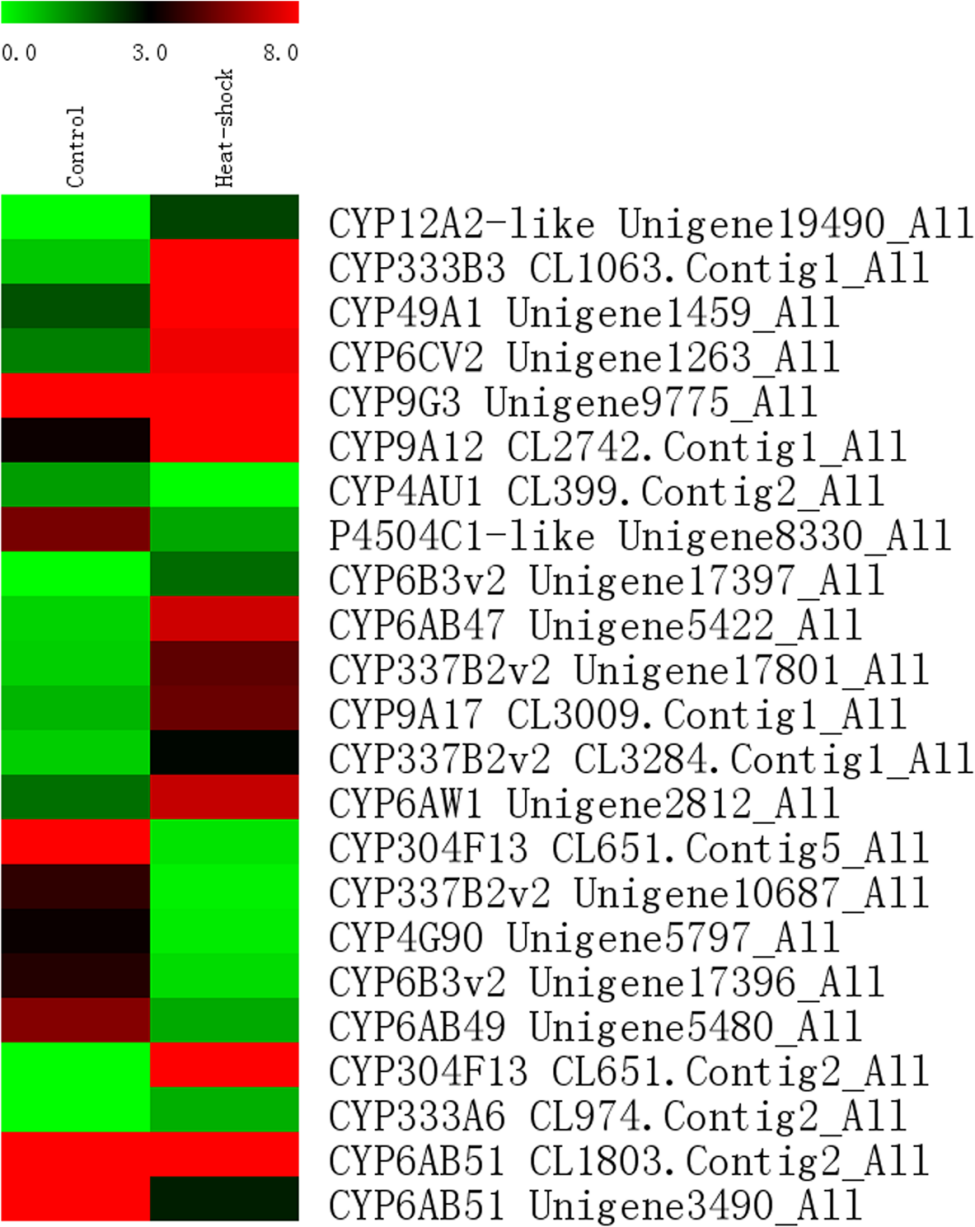
Heatmap of the different expression of cytochrome P450 genes under different heat treatment temperatures. The color scale is shown at the upper left, which encompasses from the lowest (green) to the highest (red) RPKM value

Through KEGG pathway enrichment analysis, we found that many pathways related to metabolic reactions were enriched, including carbohydrate (14 pathways), lipid (15 pathways), and amino acid metabolisms (14 pathways). The genes associated with carbohydrate metabolism were partially repressed, such as glycolysis/gluconeogenesis (16 DEGs, 0.96%), fructose and mannose metabolism (13 DEGs, 0.78%), starch and sucrose metabolism (18 DEGs, 1.08%), galactose metabolism (15 DEGs, 0.9%), and oxidative phosphorylation (17 DEGs, 1.02%). A repression in the processes involved in lipid metabolism, including sphingolipid metabolism (23 DEGs, 1.38%), steroid biosynthesis (13 DEGs, 0.78%), fatty acid degradation (21 DEGs, 1.26%), and glycerolipid metabolism (25 DEGs, 1.5%), was also observed. The downregulated genes in fructose and mannose metabolism, glycolysis/gluconeogenesis, fatty acid biosynthesis, and fatty acid elongation pathways are illustrated in Fig. 6. Significant changes in metabolism-related genes involved in heat response were consistent with a previous report describing global gene expressions in livestock [26, 27]. In addition, unigenes, which related to protein turnover processes, were induced in response to heat stress. Among these unigenes, one proteasome-related unigene (CL2694.Contig1_All) was significantly upregulated. In addition, 34 of 57 ubiquitin system-related unigenes were specifically induced in response to heat stress, suggesting that the stress-related autophagy mechanisms were induced after heat stress. 8 of 10 ribosomal proteins, which were involved in protein translation and regulation of protein formation, were upregulated. These results suggested that protein turnover response was induced for protection against the detrimental effects of protein misfolding.

### Immune response

**Fig. 7:**
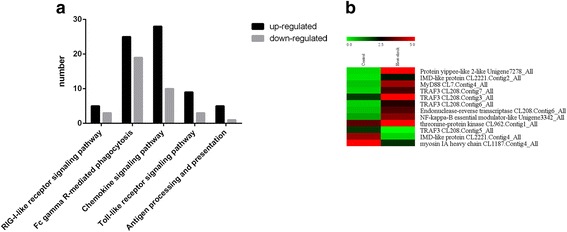
General statistics on gene regulation under different temperatures and heatmap of the expression level of Toll-like receptor signaling pathways. **a** Numbers of upregulated and downregulated genes associated with various immune events. **b** Heatmap of genes related to Toll-like receptor signaling pathways. The color scale is shown at the upper left, which encompasses from the lowest (green) to the highest (red) RPKM value

Insects lack adaptive immunity and rely on innate immune reactions for their defense [28]. In the present study, a large number of immune-related genes were enriched, and most of the gene sets demonstrated an upregulated expression after treatment at 40 °C. These genes were mainly involved in five pathways, namely, RIG-I-like receptor signaling pathway (8 DEGs, 0.48%), Fc gamma R-mediated phagocytosis (44 DEGs, 2.64%), chemokine signaling pathway (38 DEGs, 2.28%), Toll-like receptor signaling pathway (12 DEGs, 0.72%), and antigen processing and presentation (6 DEGs, 0.36%) (Fig. 7a). Nine unigenes involved in the Toll-like receptor signaling pathway were upregulated and thus may play a central role in relaying intracellular immune signals (Fig. 7b). Meanwhile, some unigenes participating in immune defense mechanisms were also detected in our data, although they do not belong to the pathways mentioned previously. Three unigenes (CL1248.Contig8_All, Unigene8334_All, and Unigene6267_All) were found to encode lectin, which is directly associated with the component of the immune system. Five genes encoding lysozyme were also determined, and three of them (CL623.Contig1_All, Unigene6122_All, and Unigene5037_All) had high expression levels and two (Unigene12321_All and CL623.Contig2_All) had low expression levels. Furthermore, some antiviral-related genes, such as genes encoding *hdd1* (Unigene19527_All), trypsin-like serine proteinase (CL1159.Contig4_All), and scavenger receptor class B (Unigene7913_All), were induced. The abundant expression of immune response-related genes suggested that the immune responses were activated after heat shock.

### Stress signal transduction

Stress signal transduction is the most important aspect for high temperature response. Many pathways involved in stress signal transduction were enriched in our analysis, such as Phosphatidylinositol (PI) signaling system (25 DEGs, 1.5%), Notch signaling pathway (16 DEGs, 0.96%), TNF signaling pathway (21 DEGs, 1.26%), Phospholipase D signaling pathway (34 DEGs, 2.04%), Jak-STAT signaling pathway (15 DEGs, 0.9%) and AMPK signaling pathway pathways (26 DEGs, 1.56%) (Table 3). The threonine protein kinase (CL962.Contig1_All) was also upregulated, and its activation was responsible for cellular stresses, such as heat shock [29]. These findings indicate the importance of signal transduction in the thermal tolerance of *G. pyloalis.*

**Table 3:** Significantly enriched signal pathways after heat treatment

Signal pathway	DEGs genes with pathway annotation	All genes with pathway annotation	*P* value	Pathway ID
Phosphatidylinositol signaling system	25 (1.5%)	162 (1.04%)	0.03917114	ko04070
Notch signaling pathway	16 (0.96%)	94 (0.6%)	0.04112893	ko04330
TNF signaling pathway	21 (1.26%)	134 (0.86%)	0.04798497	ko04668
Phospholipase D signaling pathway	34 (2.04%)	239 (1.54%)	0.05239752	ko04072
Jak-STAT signaling pathway	15 (0.9%)	91 (0.59%)	0.05950994	ko04630
AMPK signaling pathway	26 (1.56%)	182 (1.17%)	0.07817966	ko04152

### Validation of data through quantitative real-time PCR (qRT-PCR)

**Fig. 8:**
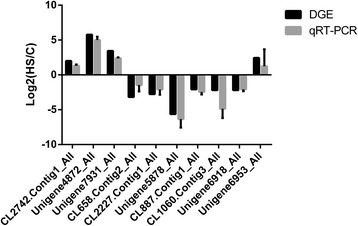
qRT-PCR analysis of the expression levels of ten unigenes. X axis represents different unigenes. CL2742.Contig1_All, cytochrome P450 9G3; Unigene4872_All, HSP 19.7; Unigene7931_All, HSP 16.1; CL658.Contig2_All, aldehyde dehydrogenase family of seven members A1; CL2227.Contig1_All, acetyltransferase 1; Unigene5878_All, hemolymph juvenile hormone-binding protein; CL887.Contig1_All, aldose 1-epimerase-like; CL1060.Contig3_All, 17-beta-hydroxysteroid dehydrogenase; Unigene6918_All, Hematopoietically-expressed homeobox protein HHEX homolog; Unigene6953_All, cytochrome P450 B5. Y axis represents the relative expression levels of genes. *Ribosomal protein L32* (*Rpl32*) was used as an internal control

Thousands of genes showed significantly different expression levels. In this study, we randomly selected ten genes to confirm their expression levels through qRT-PCR. Expression patterns were validated among the ten annotated transcripts (CL2742.Contig1_All, Unigene4872_All, Unigene7931_All, CL658.Contig2_All, CL2227.Contig1_All, Unigene5878_All, CL887.Contig1_All Unigene6953_All, Unigene6918_All and CL1060.Contig3_All). Some genes related to heat tolerance showed upregulated expression levels at 40 °C. These genes included genes encoding CYP9G3 (CL2742.Contig1_All), CYPB5 (Unigene6953_All), HSP19.7 (Unigene4872_All), and HSP16.1 (Unigene7931_All). Meanwhile, we found that *Ribosomal protein L32* (*Rpl32*) and *β-actin* were stable. The *Rpl32* was used as an internal control. The changing trend of the qRT-PCR results was consistent with the results obtained by DEG expression profiling (Fig. 8).

## Discussion

Many insects develop various mechanisms, including morphological and physiological adaptations, to cope with high-temperature stress. In this study, the morphological features of *G. pyloalis* indicated heat tolerance superior to that of *B. mori* and thus can contribute to its effective survival under high temperature. The adaptation strategies of *G. pyloalis* to heat stress may also be related to the expression of some unique genes. We conducted high-resolution analysis on transcriptome dynamics to identify the genes associated with excellent heat tolerance of *G. pyloalis*. In total, we obtained 2497 DEGs (1275 upregulated and 1222 downregulated) under heat stress and analyzed a large suite of biomarkers related to antioxidant, detoxication, metabolism, protein turnover, immune, and stress signal transduction. The GO analysis results showed that most of the DEGs after heat treatment were significantly represented in “response to stimulus”, “regulation of response to stimulus”, “response to steroid hormone” and “cellular response to steroid hormone stimulus”. This finding suggested that most of the DEGs at the early heat stress stages were mainly associated with stress response and similar experiment results were observed in the plant response to heat stress. Many genes associated with “membrane”, which revealed that most of the cells required being repaired [30]. Moreover, many highly represented pathways, such as sphingolipid metabolism, phenylalanine metabolism, metabolic pathways, and PI signaling system, were enriched when exposed to heat stress, indicating the importance of these pathways in establishing thermal tolerance [31].

In insects, HSPs act as molecular chaperones and participate in various cellular processes, such as protein folding, proteolytic pathway, or stabilizing denatured proteins [32–34]. HSPs can be divided into several families, namely, HSP40, HSP60, HSP70, HSP90 and sHSPs according to their molecular weights [35]. Twelve HSPs distributed in the HSP70, HSP40, and sHSPs were observed to respond to heat treatment during the transcriptome analysis of *G. pyloalis*. The preferential induction of HSPs upon heat stress has been widely reported in numerous studies. In *D. melanogaster*, the accumulation of a major HSP, namely, HSP70, affects inducible thermal tolerance in larvae and pupae [36]. In *Ceratitis capitata,* expression of Hsp70 and thermal tolerance were assayed at a range of temperatures in several stages of its development. In the current study, the induced expression of five HSP70s in *G. pyloalis* possibly facilitated the refolding of damaged proteins and prevented protein aggregation under heat stress. In addition, five small HSPs of *G. pyloalis* were upregulated by heat stress and likely cooperated with co-chaperones for the prevention of cellular protein aggregation [37]. sHSPs are abundant in nearly all organisms and act as a response machine of organisms to some extreme stresses [38]. These results suggested that the induction of HSP was an evolutionarily conserved mechanism, and the coordinate upregulation of the molecular chaperones, including HSP70 and sHSP, was an important factor in acquiring thermal tolerance and can render the midgut cells of *G. pyloalis* resistant to heat stress.

Exposure to heat stress can result in ROS production, which can directly lead to a variety of toxic effects, including protein dysfunction, lipid peroxidation, and oxidative stress. In relation to signal transduction of ROS, we detected upregulation of several genes involved in vitamin digestion and absorption pathways. Many studies have demonstrated the importance of vitamins in oxidative stress responses. Sies [39] mentioned that vitamins C and E can react with free radicals and singlet molecular oxygen, which is the basis for their functions as antioxidants. Notablely, the induction of vitamin digestion and absorption pathway may suggest a specific function in the adaption of insect to high temperature. To the best of our knowledge, this result is some of the first evidence indicating the existence of vitamin digestion and absorption pathway after heat stress in insects. In Table 2, several antioxidant-related genes, including superoxide dismutase (SOD), peroxidase (POD), and thioredoxin were strongly upregulated after 40 °C treatment. Recent proteomic analysis showed that SOD overexpression upregulated ROS scavenging in rice grains under heat stress. The SOD knockout rice was susceptible to heat stress, which suggested that SOD played an important role in adaptation to heat stress [40]. POD, as a regulator of ROS scavenger, is a hematin-containing oxidase that can catalyze the oxidation of reduced compounds [41]. Meanwhile, thioredoxin can be potentially responsive to ROS and can catalyze electron transport to ribonucleotide reductase and other reductive enzymes and transcription factors [42]. The coordinate upregulation of the three antioxidant-related genes after 40 °C treatment suggested that they were regulators of ROS scavenger in *G. pyloalis* and were potentially generate ROS in response to heat stress. Similar antioxidant-related genes were also observed in *Bactrocera dorsalis* and *Bemisia tabaci* exposed to high temperatures [43, 44]. Our data indicated that a link between oxidative stress and vitamin digestion and absorption was present in heat tolerance of *G. pyloalis.*

In addition to ROS generation, heat stress can promote toxic substance accumulation. In this study, we found that many detoxication-related genes, such as genes coding CYP, glutathione-S-transferase (GST), and ALDH, showed upregulated mRNA expression patterns after 4 h of heat stress. In most species, CYP effectively catalyzes lipid peroxidation. Meanwhile, the induction of a state of oxidative stress plays a central role in CYP-dependent cytotoxicity [45]. Apart from eliminating drugs, cytochrome P450s catalyze a broad range of oxidative processes involved in the metabolism of fatty acids and biosynthesis of sterols. Therefore, the induction of 14 CYPs in *G. pyloalis* may be an essential step in heat tolerance. In addition, we found 3 of 5 CYPs in *B. mori* were significantly induced at 40 °C, suggesting several CYPs played a conserved role in response to heat stress (Additional file 6). However, further experiments are necessary to determine the exact function of these proteins. Meanwhile, GSTs comprise a family of isozymes and can convert toxic substances into reduced toxic metabolites through chemical reactions involving the conjugation of glutathione [46]. They protect macromolecules from reactive electrophiles, such as environmental carcinogens, ROS products, and chemotherapeutic agents [46]. Our observation suggested that the upregulation of genes encoding GST was related to increased intracellular oxidative stress and cellular toxic substance metabolism [47]. Detoxification pathways identified here provided new insights for the investigation of the molecular mechanisms of heat stress tolerance. ALDHs can eliminate toxic aldehydes by catalyzing their oxidation to nonreactive acids, the enzyme activities of which is mainly implicated in the metabolism of endogenous lipid peroxidation products [48]. Two mRNAs for ALDH were significantly upregulated after heat shock, suggesting they contribute to the improved heat tolerance through metabolic pathway.

Insects have developed a range of strategies, such as metabolism adaptation, to manage heat stress. Our results indicated that the metabolism of *G. pyloalis* in high temperature was weak. A similar reduction was observed in livestock [27]. The suppression of metabolism progress may be a conservative progress and can reflect cellular homeostasis or an energy-saving mechanism to manage heat stress, because ATP generation is a highly-regulated process involving three metabolism pathways, namely, glycolysis, tricarboxylic acid cycle, and oxidative phosphorylation. In addition, the ROS generated by heat stress can chemically modify proteins and alter their biological functions. Therefore, the removal of damaged proteins is vital for the maintenance of cellular homeostasis. Numerous studies have revealed that the ubiquitin-proteasome system plays a vital role in recognizing and degrading damaged proteins [49, 50]. In the present study, 34 ubiquitin-related unigenes were induced by heat stress. These results revealed that the upregulation of protein degradation progress was involved in stress response and adaptation.

For insects, the innate immune system is the major effector response system [51]. In our results, the activities of the RIG-I-like and Toll-like receptor signaling pathways were modulated by heat stress. The role of transcriptional activation of the Toll-like receptor in heat shock response has been reported [52–54]. Several reports have described some endogenous ligands, such as HSP60 and HSP90, that activate TLR4 to regulate innate immune responses and are also induced by high temperature [55]. Frequent confrontation of the immune system with HSPs can potentiate immunity. The upregulation of lectin, lysozyme, *Hdd1*, trypsin-like serine proteinase, and scavenger receptor class B under high temperatures further suggested that heat stress facilitated the components of immune defense.

The sensing and transduction of intracellular stress signal are critical for the adaptation and survival of insects under heat stress. In plants, the involvement of calcium and calcium-activated calmodulin in heat shock signal transduction was already investigated [56]. However, the mechanisms that enable insects to sense heat signal and trigger intracellular responses remain unclear. The PI signaling system is vital during the progress of plant development and growth and associated with cellular responses to environmental stress [57]. Meanwhile, Notch signaling pathway participates in physiological and stress erythropoiesis [58]. In this study, the preferential induction of PI signaling system and Notch signaling pathway under heat stress was uncovered and the results suggested that gene expression in the PI signaling system was related to the heat tolerance mechanism of *G. pyloalis*. These results suggested that the PI signaling system and Notch signaling pathway rapidly and effectively regulated downstream signaling and gene expression in *G. pyloalis* in response to high-temperature stress.

## Conclusions

In the present study, we presented the first comprehensive evaluation of the midgut transcriptome of *G. pyloalis* by using Illumina sequencing technology and conducted a comparative expression analysis after heat treatment. A total of 34,034 unigenes, which might provide a major genomic resource for investigating the midgut of *G. pyloalis*, were obtained*.* Most of these genes had an annotation with matches in the seven functional databases. Furthermore, 2497 DEGs were identified in the midgut after heat stress. The results of the GO and KEGG pathway enrichment analyses indicated that the DEGs related to metabolism, immunity, and signal transduction were enriched after heat stress. In addition, the transcriptome results revealed a number of genes that are potentially relevant to HSPs, antioxidant, and detoxication in *G. pyloalis*. These genes could be major targets for thermal tolerance. Many of which can be understood with deep functional studies. Nonetheless, our study provides insight into the heat tolerance of insects.

## Methods

### Biological materials and tissue collection

*G. pyloalis* larvae were collected from the mulberry fields of Zhejiang University, Hangzhou (120.2′ E, 30.3′ N), Zhejiang Province, China. *B. mori* (N4) larvae were reared as described previously [59]. The method of high temperature treatments or thermal exposure was modified from methods of Xiao et al. (2016), Li et al. (2014) and Moallem et al. (2017) [20, 60, 61]. *G. pyloalis* and *B. mori* treated at 25 °C served as controls. For treatments, larvae on day 3 of the fifth instar were selected and separated into two groups with 13 larvae each. Larvae were reared in different chambers at 25 °C and 40 °C for 4 h, respectively. They were supplied hourly with fresh mulberry leaves. Three biological replications were performed with each treatment. Four males and four females were selected for each treatment. Clean midgut samples were collected from eight (four males and four females) *G. pyloalis* and *B. mori*, respectively. The sample were freezed at −80 °C immediately.

### Histological staining

After being exposed to different temperature treatments as described above, the samples of larval *G. pyloalis* and *B. mori* were collected for hematoxylin–eosin (HE) staining. The midgut of *G. pyloalis* was dissected and fixed in formalin-acetic acid-alcohol (FAA) liquid for 12 h. The samples were dehydrated through a series of graded ethanol baths (70%–100%) to displace water, and then infiltrated with paraffin wax, a finally cut into sections (5 μm). The obtained tissue sections were stained with 2% Mayer’s hematoxylin and 1% eosin [62]. Slides were observed and image can be examined directly in the microscope (Nikon Nis-Elements).

### De novo assembly and gene annotation

Total RNA was extracted using RNAiso plus reagent (TaKaRa) according to the manufacturer’s protocol. The concentration and quality of total RNA was quantified using NanoDrop Spectrophotometer (Thermo Fisher Scientific). For each group, total RNA from three replicates was pooled together in equal quantities. Approximately 6 μg of RNA representing each group were used for illumine sequence. After the high-throughput sequencing (Illumina HiSeq 4000) of midgut samples, we used Trinity to filter raw reads by discarding low-quality, adaptor-polluted, and high-content unknown base reads and to generate 150 bp paired-end read lengths. The clean reads were assembled into primary transcripts, and overlapping transcripts were assembled into large contigs after removing redundant transcripts. The final unigenes were obtained through gene family clustering with Tgicl [63]. Gene function was annotated based on the following databases: NCBI non-redundant protein sequences (NR); InterPro member database; Clusters of Orthologous Groups of proteins (KOG/COG); Swiss-Prot (a manually annotated and reviewed protein sequence database); Kyoto Encyclopedia of Genes and Genomes (KEGG); and Gene Ontology (GO). The unigenes that were not aligned to any database mentioned above were predicted by ESTScan (http://sourceforge.net/projects/estscan).

### Gene expression quantification

Bowtie2 software (http://bowtie-bio.sourceforge.net/Bowtie2/index.shtml) was used to map all the clean reads to the unigene library. Gene expression level was then calculated using the RSEM software (http://deweylab.biostat.wisc.edu/RSEM). The fragments per kilobase of transcript per million (FPKM) mapped reads can be used to quantify the unigene expression level. False discovery rate (FDR) was used to determine the threshold *P*-value in multiple tests. Furthermore, |fold change| ≥ 4.0 and FDR ≤ 0.001 were used as the parameters for determining significant differences in gene expression. The DEGs were then used for GO and KEGG enrichment analyses. The *P*-value calculating formula in the hypergeometric test is:

$$ P=1-\sum \limits_{i=0}^{\mathrm{m}-1}\frac{\left(\begin{array}{c}M\\ {}\mathrm{i}\end{array}\right)\left(\begin{array}{c}N-M\\ {}n-i\end{array}\right)}{\left(\begin{array}{c}N\\ {}n\end{array}\right)} $$

In this equation, N and n indicate the number of genes with GO/KEGG annotations and the number of DEGs in N, respectively. The variables M and m represent the numbers of genes and DEGs, respectively, in each GO/KEGG term.

### qRT-PCR validation

Ten annotated unigenes and five *B. mori* CYPs were randomly selected to quantify by qRT-PCR and evaluate data level. RNAiso plus reagent (TaKaRa) was used to extract total RNAs from three biological replications, and Primer Premier 5.0 was used to perform the primer design. qRT-PCR primers are displayed in Additional file 7 and Additional file 8. The reverse transcription system was used to synthesize cDNA as described previously [64–66]. qRT-PCR was performed on a LightCycler® 480 real-time PCR system (Roche). The reaction system consisted of 2 μl of cDNA, 10 μl of SYBR Green qPCR master mix (TaKaRa), 0.5 μl of each of the primers, and sterile water made up to 20 μl. The ΔΔCt method was used to analyze the relative differences in transcript levels [67]. *Ribosomal protein L32* (*Rpl32*) was used for internal control, and the experiment was performed on three biological replicates.

## Additional files

Additional file 1: Distribution of base content and quality. (DOCX 18 kb)

Additional file 2: Unigene length distribution in *G. pyloalis* midgut with different treatments. X axis represents the length of unigenes. Y axis represents the number of unigenes. A: “Control” unigene length distribution. B: “Heat shock” unigene length distribution. (TIFF 1807 kb)

Additional file 3: Distribution of annotated species. (TIFF 5077 kb)

Additional file 4: List of the significantly up- and down-regulated genes. (XLSX 570 kb)

Additional file 5: Pathway functional enrichment of DEGs. Based on the KEGG results, the metabolic pathway was the most enriched KEGG pathway under heat stress. (TIFF 1458 kb)

Additional file 6: qRT-PCR analysis of the expression levels of five randomly selected *B. mori* genes. (TIFF 61 kb)

Additional file 7: Primers used for *B. mori* CYPs. (DOCX 15 kb)

Additional file 8: Primers used for *G. pyloalis*. (DOCX 19 kb)

## Abbreviations

17 beta-HSD: 17-beta-hydroxysteroid dehydrogenase
A1E: aldose 1-epimerase-like
ALDH: Aldehyde dehydrogenase
AT: acetyltransferase 1
BLAST: Basic local alignment search tool
COG: Clusters of Orthologous Groups
CYP: cytochrome P450
DEGs: Differentially expressed genes
FAA: formalin-acetic acid-alcohol
FPKM: Fragments Per Kilobase of transcript per Million
GO: Gene ontology
GST: glutathione-S-transferase
HE: hematoxylin–eosin
Hhex: Hematopoietically-expressed homeobox protein
HSP: heat shock protein
JHBP: hemolymph juvenile hormone-binding protein
KEGG: Kyoto Encyclopedia of Genes and Genomes
NCBI: National Center for Biotechnology Information
NR: NCBI non-redundant protein database
Nt: Nucleotide sequence database
PI: Phosphatidylinositol
POD: peroxidase
qRT-PCR: Quantitative real-time PCR
RNA-Seq: RNA-sequencing
ROS: Reactive oxygen species
Rpl32: Ribosomal protein L32
SOD: superoxide dismutase
Swiss-Prot: UniProtKB/Swiss-Prot
TCA: tricarboxylic acid cycle
