# Supplementary material for: Comparative transcriptome analysis of Glyphodes pyloalis Walker (Lepidoptera: Pyralidae) reveals novel insights into heat stress tolerance in insects
Source: BMC Genomics. 2017 Dec 19;18:974. doi: 10.1186/s12864-017-4355-5 (PMC5735938; doi:10.1186/s12864-017-4355-5)
Supplement: Supplementary file 1 — Distribution of base content and quality. (DOCX 18 kb) [file 12864_2017_4355_MOESM1_ESM.docx]

**Additional file 1.** The distribution of base content and quality.

| Sample | Control | Heat-shock |
| --- | --- | --- |
| Total Raw Reads (Mb) | 47.36 | 48.99 |
| Total Clean Reads (Mb) | 44.57 | 44.53 |
| Total Clean Bases (Gb) | 6.69 | 6.68 |
| Clean Reads Q20 (%) | 96.28 | 96.28 |
| Clean Reads Q30 (%) | 91.81 | 91.84 |
| Clean Reads Ratio (%) | 94.10 | 90.89 |

Q20/Q30: the rate of bases which quality is greater than 20/30
