# Supplementary figures and images for: Comparative transcriptome analysis of Glyphodes pyloalis Walker (Lepidoptera: Pyralidae) reveals novel insights into heat stress tolerance in insects
Source: BMC Genomics. 2017 Dec 19;18:974. doi: 10.1186/s12864-017-4355-5 (PMC5735938; doi:10.1186/s12864-017-4355-5)

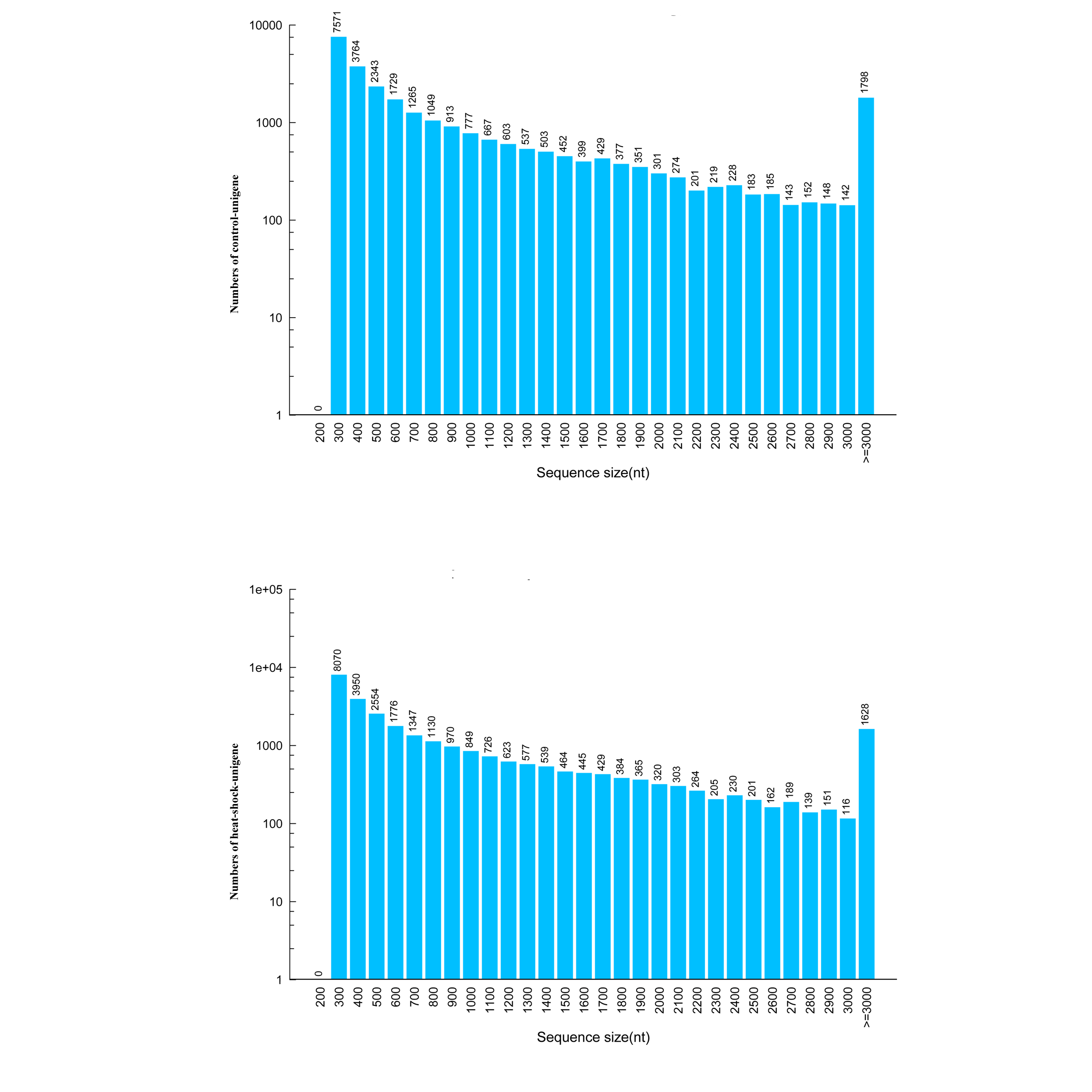

Supplement: Supplementary file 2 — Unigene length distribution in G. pyloalis midgut with different treatments. X axis represents the length of unigenes. Y axis represents the number of unigenes. A: “Control” unigene length distribution. B: “Heat shock” unigene length distribution. (TIFF 1807 kb) [file 12864_2017_4355_MOESM2_ESM.tif]

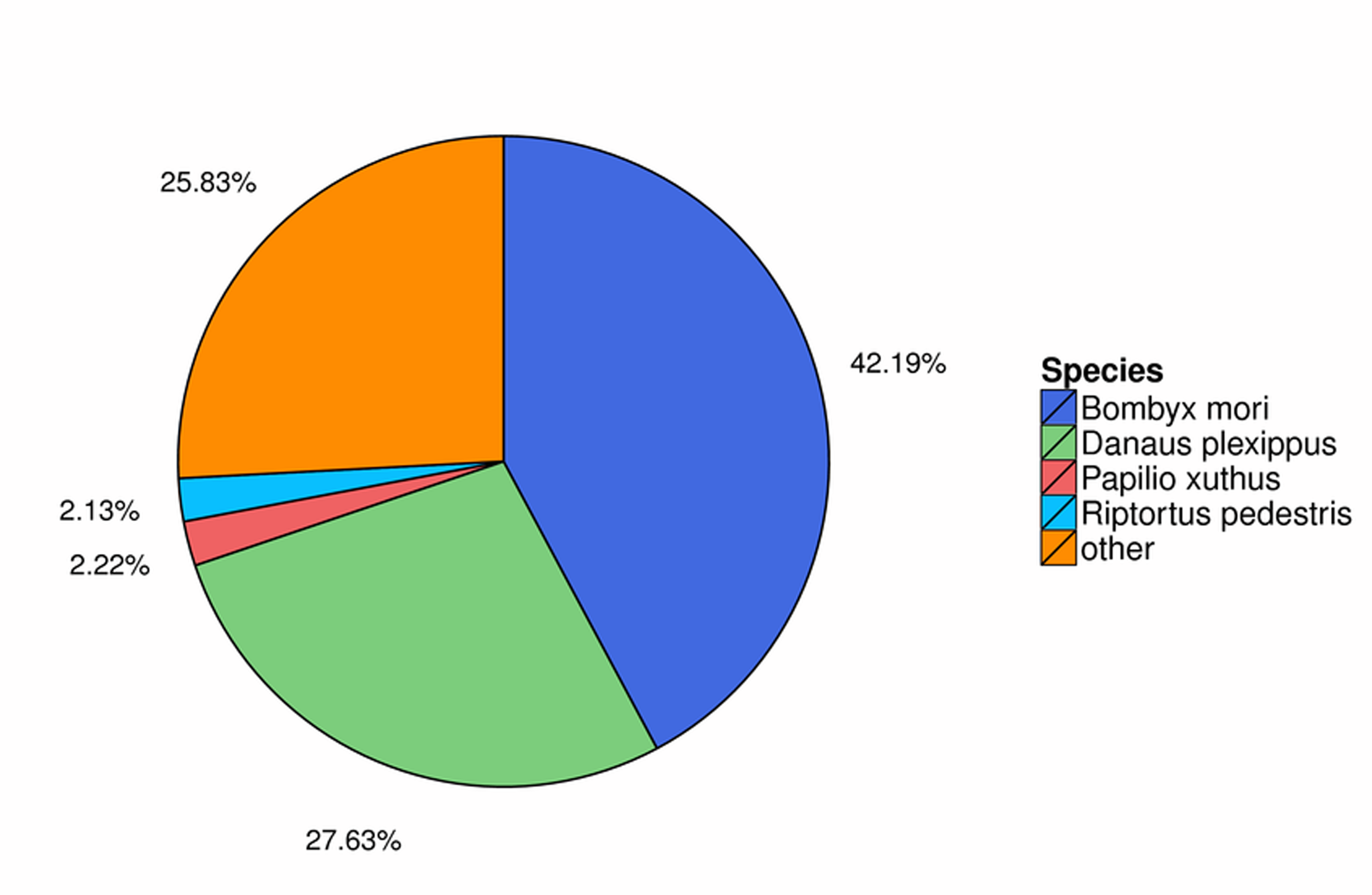

Supplement: Supplementary file 3 — Distribution of annotated species. (TIFF 5077 kb) [file 12864_2017_4355_MOESM3_ESM.tif]

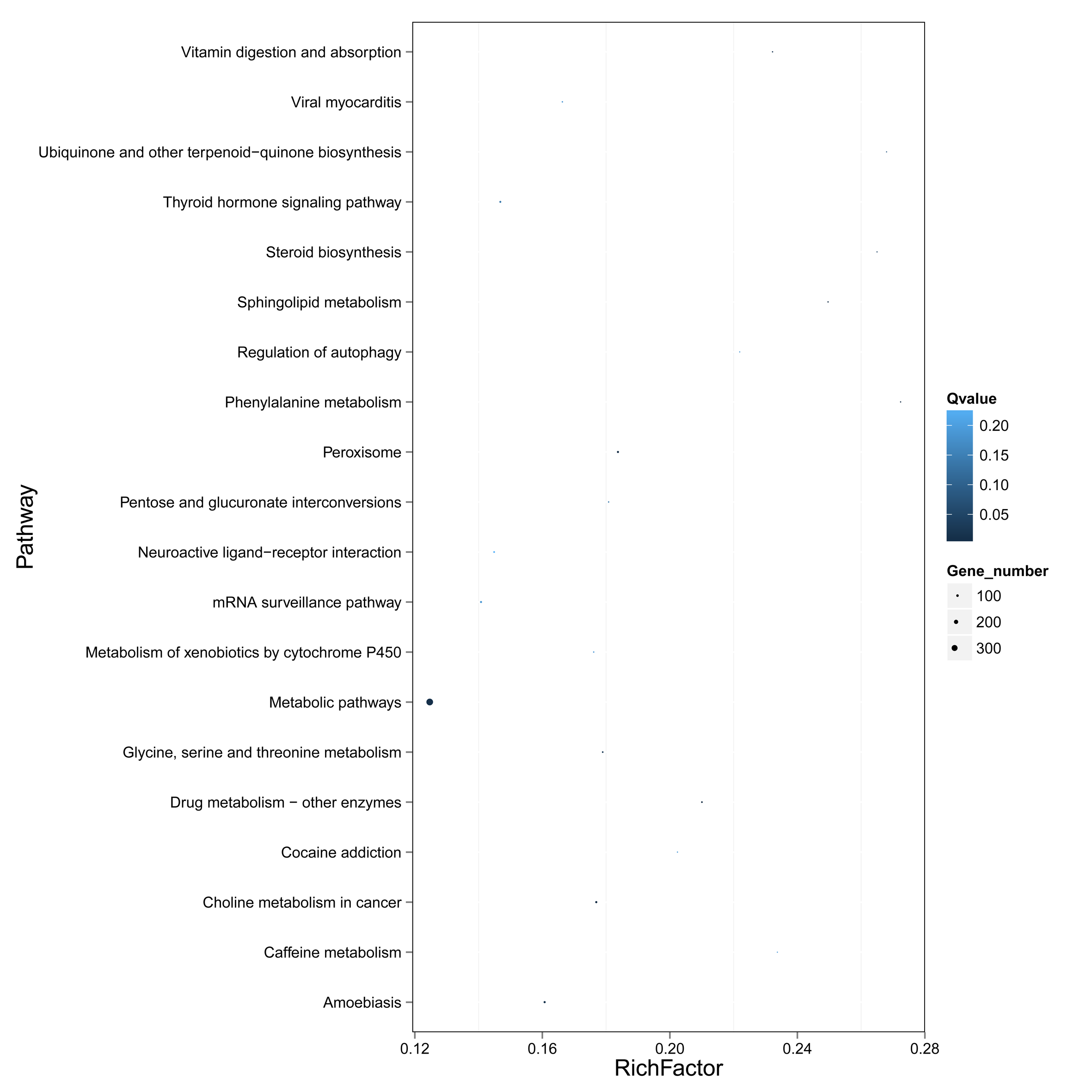

Supplement: Supplementary file 5 — Pathway functional enrichment of DEGs. Based on the KEGG results, the metabolic pathway was the most enriched KEGG pathway under heat stress. (TIFF 1458 kb) [file 12864_2017_4355_MOESM5_ESM.tif]
