# Supplementary material for: Comparative transcriptome analysis of Glyphodes pyloalis Walker (Lepidoptera: Pyralidae) reveals novel insights into heat stress tolerance in insects
Source: BMC Genomics. 2017 Dec 19;18:974. doi: 10.1186/s12864-017-4355-5 (PMC5735938; doi:10.1186/s12864-017-4355-5)
Supplement: Supplementary file 7 — Primers used for B. mori CYPs. (DOCX 15 kb) [file 12864_2017_4355_MOESM7_ESM.docx]

**Additional file 7.** Primers used for *B. mori* CYP qRT-PCR

| **Gene** | ***G. pyloalis* Gene ID** | ***B. mori* Gene ID** | | **Identify** | | **Primer sequence (5’-3’)** |  |
| --- | --- | --- | --- | --- | --- | --- | --- |
| *CYP12A2* | Unigene19490_All | | XM_012695536 | | 62% | TCCGCAGCCCGAAGAGTT  TGCTATCCGACGCCCAAT | |
| *CYP12A2Like*  *CYP9A22*  *P450*  *CYP333B1* | CL1063.Contig1_All  Unigene9775_All  Unigene2812_All  CL974.Contig2_All | | XM_004927911  NM_001102464  NM_001309591  AK289309 | | 55%  63%  75%  36% | AACAGGATACGTGGTCAA  TCCAAATACTGGGCTTGA  AGTTGGCTCTAAACCCTG  TCCACAATCTAAGTACCTCC  CGCCCAACACTTTCCTAA  GACAACTCCTCGGTCCTT  GTTGAGGGAAGAAGTGATG  TGTACTCCTTTGTGGTGC | |
