# Supplementary material for: Comparative transcriptome analysis of Glyphodes pyloalis Walker (Lepidoptera: Pyralidae) reveals novel insights into heat stress tolerance in insects
Source: BMC Genomics. 2017 Dec 19;18:974. doi: 10.1186/s12864-017-4355-5 (PMC5735938; doi:10.1186/s12864-017-4355-5)
Supplement: Supplementary file 8 — Primers used for G. pyloalis. (DOCX 19 kb) [file 12864_2017_4355_MOESM8_ESM.docx]

**Additional file 8**. Primers used for qRT-PCR.

| **Gene name** | **Gene ID** | **Pvalue** | **FDR** | **Primer sequence (5’-3’)** |  |
| --- | --- | --- | --- | --- | --- |
| *CYP 9G3* | CL2742.Contig1_All | 1.683306E-25 | 2.65E-24 | CGCTCCGTCTGTCACT  CTGGTCGTTCTATCTTGTTC | |
| *hsp19.7*  *hsp16.2*  *ALDH*  *AT*  *JHBP*  *A1E*  *17 beta-HSD*  *hhex*  *CYP B5*  *rpl32*  *beta actin* | Unigene4872_All  Unigene7931_All  CL658.Contig2_All  CL2227.Contig1_All  Unigene5878_All  CL887.Contig1_All  CL1060.Contig3_All  Unigene6918_All  Unigene6953_All  CL762.Contig1_All  CL212.Contig1_All | 0  0  0  1.00E-305  2.98E-195  1.49E-64  5.61E-25  2.72E-38  0  1.06E-118  0.214983415 | 0  0  0  1.48E-303  2.72E-193  4.85E-63  8.69E-24  5.85E-37  0  5.94E-117  0.308844 | CCAGTATCAAGTCGGACAAAGA  GCTTCCCTTCCACCACTATG  GGAACCCTTCAGGCAACA  CGACGGCAAGAAAGTGGA  TGAAGGCGGTGATGATGC  TTTGCGACCTGGCTCTGG  GACCGCTGAGAAGTTGGG  CGGCTACGCTGACTTGCT  CTTAGCAAAGGCAAGCAGG  ATGGGCAGCACCAAGAGGC  GACTTTGGCACCTACCGC  CATCTGCTATTACTCCGTTC  GTGGGAGGCTATGCTTGA  CACGGGACTCTGCTGAAA  TGAACGGGTGAGATACAAA  GAACTAAGGAACAAGCAGGA  ACCAGGTTCATCATCCACA  CTCGTGCTTGTCCTCCTC  CGATCACCTTCCGCTTCT  TGCTACCCAATGGCTTCC  GGGTGTCATGGTCGGTATGG  CATGTCGTCCCAGTTGGTGA | |

FDR, false discovery rate, the terms which FDR not larger than 0.001 are defined as significant enriched. CYP 9G3, cytochrome P450 9G3; hsp19.7, 19.7-kDa heat shock protein; hsp16.1, 16.1-kDa heat shock protein; ALDH, aldehyde dehydrogenase; AT, acetyltransferase 1; JHBP, hemolymph juvenile hormone-binding protein; A1E, aldose 1-epimerase-like; 17 beta-HSD, 17-beta-hydroxysteroid dehydrogenase; hhex, Hematopoietically-expressed homeobox protein; CYP B5, cytochrome P450 B5. rpl32, Ribosomal protein L32.
